# Diamond beamline I07: a beamline for surface and interface diffraction

**DOI:** 10.1107/S1600577516009875

**Published:** 2016-07-27

**Authors:** Chris Nicklin, Tom Arnold, Jonathan Rawle, Adam Warne

**Affiliations:** a Beamline I07, Diamond Light Source Ltd, UK

**Keywords:** X-ray diffraction, surface, structure, reflectivity, grazing incidence, surface structure, interfaces, X-ray scattering

## Abstract

Beamline I07 at Diamond Light Source is dedicated to the study of the structure of surfaces and interfaces for a wide range of sample types, from soft matter to ultrahigh vacuum. The beamline operates in the energy range 8–30 keV and has two endstations. The first houses a 2+3 diffractometer, which acts as a versatile platform for grazing-incidence techniques including surface X-ray diffraction, grazing-incidence small- (and wide-) angle X-ray scattering, X-ray reflectivity and grazing-incidence X-ray diffraction. A method for deflecting the X-rays (a double-crystal deflector) has been designed and incorporated into this endstation, extending the surfaces that can be studied to include structures formed on liquid surfaces or at liquid–liquid interfaces. The second experimental hutch contains a similar diffractometer with a large environmental chamber mounted on it, dedicated to *in situ* ultrahigh-vacuum studies. It houses a range of complementary surface science equipment including a scanning tunnelling microscope, low-energy electron diffraction and X-ray photoelectron spectroscopy ensuring that correlations between the different techniques can be performed on the same sample, in the same chamber. This endstation allows accurate determination of well ordered structures, measurement of growth behaviour during molecular beam epitaxy and has also been used to measure coherent X-ray diffraction from nanoparticles during alloying.

## Abstract 2

A beamline is described for grazing-incidence X-ray diffraction at Diamond Light Source that houses two experimental hutches, the first housing a large multi-circle diffractometer on which a wide variety of sample environments can be mounted and the second with an ultrahigh-vacuum system on the diffractometer to enable *in situ* studies of molecular beam epitaxy. Techniques available include X-ray reflectivity, surface X-ray diffraction, grazing-incidence small-angle X-ray scattering, grazing-incidence wide-angle X-ray scattering and grazing-incidence diffraction.

## 1. Introduction   

The study of surfaces and interfaces continues to be of fundamental importance, as the boundary between two materials is where key interactions occur; for example, an automotive catalyst works by a gas interacting with the surface to produce less harmful products. The outer atoms at a surface have a lower coordination and different chemical environment that can lead to significant changes to the properties of the material. Correlating the structure and properties is key to understanding the origin of the changes and to developing new materials with improved or tailored functionality. A number of techniques have been developed to study surfaces but in many cases they are limited by the environment in which they can operate. Many rely on electron diffraction or spectroscopy to provide information and require an ultrahigh-vacuum (UHV) environment. Great inroads have been made using these techniques but it has been recognized that understanding the detail of the processes in more realistic operating conditions is essential. Hard X-ray diffraction is an ideal technique to probe the structure as it is not confined to UHV, allowing interfaces to be studied under a range of conditions, including under liquids, high gas pressures or buried solid–solid interfaces.

Grazing-incidence X-ray scattering methods are a powerful probe of the surface or interface structure, capable of giving very precise information about long-range order in materials. The grazing-incidence geometry provides surface sensitivity and a number of different measurements can reveal the layer structure, detailed atomic surface structure or the shape and size of nanoparticles on the sample.

## 2. Beamline overview   

Beamline I07 is a hard X-ray (8–30 keV) high-resolution diffraction beamline at Diamond Light Source (DLS), dedicated to the study of surfaces and interfaces using grazing-incidence angle X-ray scattering methods. A wide range of techniques are fully integrated into I07 including surface X-ray diffraction (SXRD), grazing-incidence X-ray diffraction (GIXD), X-ray reflectivity (XRR) and grazing-incidence small-angle X-ray scattering (GISAXS). Additionally, the beamline has been used for several other experiments including coherent X-ray diffraction. I07 is located on a 5 m straight section of the Diamond storage ring, enabling it to be sourced by a 2 m-long undulator with a minimum gap of 5 mm. The geometry of the scattering requires that an intense source of X-rays is incident on the small sample (of the order of a few millimetres diameter) at grazing angles close to the critical angle of the material (typically ∼0.2°) to enhance the surface sensitivity.

A beamline for surface and interface diffraction was initially proposed by a working group (established in 2002) as part of the Phase 2 development at DLS. This phase of the project enabled the development of 15 beamlines, to add to the seven funded during the initial construction. The beamline was prioritized by the scientific advisory committee in May 2003 and approved by DLS management and the board later that year. The detailed design started in 2005 with the appointment of the Principal Beamline Scientist, and construction began in July 2007. Beamline I07 became the 14th operational beamline at DLS when it took first users in October 2009.

### 2.1. General layout   

**Figure 1:**
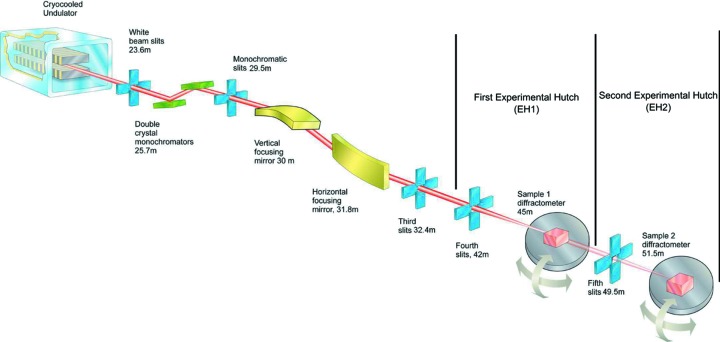
Schematic layout of beamline I07 showing some key components marked together with distance from the source.

The general layout and positions of the major optical components of the beamline are shown in Fig. 1 ▸ which highlights the two-hutch in-line design of the beamline. This approach enables experiments to be prepared in the rear hutch (EH2) while X-ray measurements are going on in the upstream hutch (EH1). EH2 is dedicated to studies requiring *in situ* UHV sample preparation that can take many days to achieve the desired cleanliness or structure to be investigated. Fig. 1 ▸ shows only the major components; in addition there is other equipment such as diagnostics, calibration foils and attenuators, details of which are given in Table 1 ▸.

### 2.2. Undulator   

**Figure 2:**
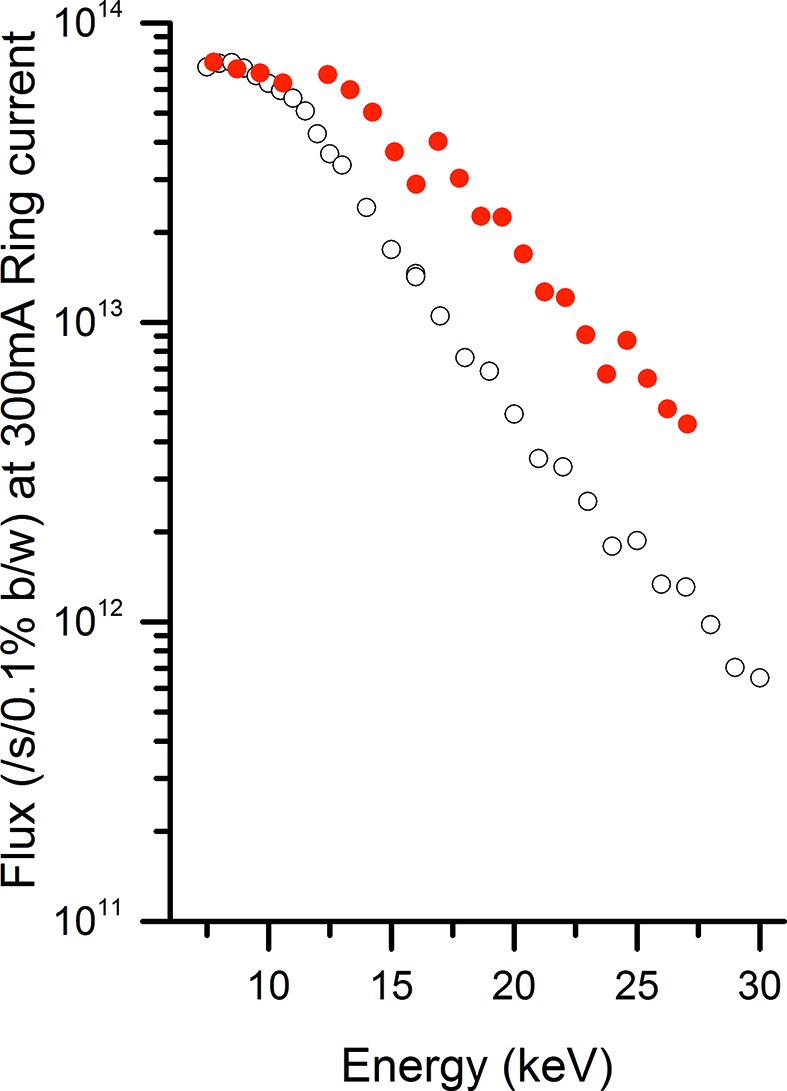
Flux from the current 17.7 mm-period CPMU (filled circles) in comparison with a 23.0 mm in-vacuum undulator (unfilled circles).

The beamline is located at the centre of a 5 m-long straight section of the storage ring, where the source size is 122.9 µm horizontally by 6.4 µm vertically with divergence of 24.2 µrad and 4.2 µrad, respectively. I07 is sourced by a cryogenic permanent-magnet undulator (CPMU) that is cooled to 150 K using a liquid-nitrogen cryocooler. Reducing the temperature is known to increase the magnetic field and coercivity of certain permanent magnets (Hara *et al.*, 2004 ▸). In the case of I07, NdFeB permanent magnets are used and have a field that increases by ∼20% when cooled. Initially the beamline had a 23 mm-period (U23) 2 m-long undulator with a 5 mm minimum gap that gave an energy-dependent flux curve as shown in Fig. 2 ▸, as measured using a calibrated photodiode at the sample position (all beamline slits set to 1 mm × 1 mm). After replacement with a cryogenically cooled 17.7 mm-period undulator (of the same length), the flux increased up to a factor of three at the higher energies as shown in the figure.

### 2.3. Monochromator   

Energy selection in the range 6–30-keV is achieved by a commercial double-crystal monochromator (DCM) from Accel, which uses a set of two Si(111) crystals on an *in vacuum* rotation stage. Both crystals are cooled using liquid nitrogen *via* a Cryotherm cryocooler system to reduce the thermal heat bump on the sample caused by the impinging beam, and a recirculating chiller (Lauda) is used to keep other components at a constant temperature. A translation stage on the second crystal maintains the outgoing beam at a constant exit height and therefore changing energy is straightforward, by changing the Bragg angle and optimizing the gap of the insertion device to give a peak in flux at the desired energy. The energy resolution is measured to be 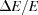 = 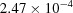, consistent with the expected values for Si(111) crystals.

### 2.4. Mirrors   

Focusing of the X-rays is achieved using a pair of bimorph mirrors mounted in a Kirkpatrick–Baez geometry, enabling independent control of the vertical and horizontal beam size. The upstream vertical focusing mirror (VFM) deflects the beam downwards, while the horizontal focusing mirror (HFM) deflects the beam outboard. Both mirrors share a common vacuum vessel with a base pressure of 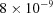
 mbar. To maximize the footprint of the beam over the length of the mirrors, the dimensions of each one have been matched to the divergence of the incident beam and a 3 mrad incidence angle. The VFM (600 mm long) is shorter than the HFM (1050 mm long) as the beam size vertically is significantly smaller than it is horizontally due to the lower divergence (31 µrad compared with 82 µrad). Both mirrors have three stripes along the full length of their surface; a bare silica in the centre with coatings of rhodium or platinum on either side. Harmonic rejection is achieved using the energy cutoff of the coating material, to prevent transmission of the higher harmonics (measured harmonic rejection rate of third order 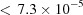). The mirror therefore acts as a low-pass filter where the cutoff energy is dependent on the coating material and X-ray incidence angle.

### 2.5. Endstations   

The first experimental hutch contains several beam-conditioning components, including slits, beam position monitors and attenuators in addition to a double-crystal deflector (DCD) that is used in the study of liquid surfaces (Arnold *et al.*, 2012 ▸). The exit window of the DCD has replaceable nose cones depending on the experiments to be undertaken, either a large Be window that allows the deflected beam from the DCD to exit the chamber or a vacuum bellows that links onto a set of slits. This section of the beamline also contains an in-vacuum fast shutter based on a cylinder with a slot in it that rotates to allow the X-ray beam through and is controlled by the detectors. This means that samples are only exposed to X-rays when the measurement is taking place, essential for many experiments where beam damage occurs such as in organic films (*e.g.* polymer photovoltaics or lipid bilayers). The final component in the beamline is a micro ion chamber (ADC) that is used as a monitor of the incident beam intensity.

The beam size at the sample position can be varied between a full width at half-maximum (FWHM) of 80 µm to 200 µm vertically and 120 µm to 400 µm horizontally. At the minimum focus the beam divergence at the sample position is 80 µrad × 55 µrad. Well calibrated attenuators are essential to increase the measurable dynamic range of the scattered signal, which can include recording intense Bragg peaks and very weak signals due solely to surface scattering. The attenuators are based around a series of 12 UHV linear actuators, eight of which hold aluminium discs with the thinnest foil of nominally 50 µm, and each subsequent one doubling in thickness. There are also three filters of molybdenum (of thickness 100, 200 and 400 µm) that are required for the higher energies available at the beamline. The final attenuator is a thick lead disc, that is used to block the beam in case of problems (*e.g.* detector overload).

#### 2.5.1. Experimental hutch 1   

**Figure 3:**
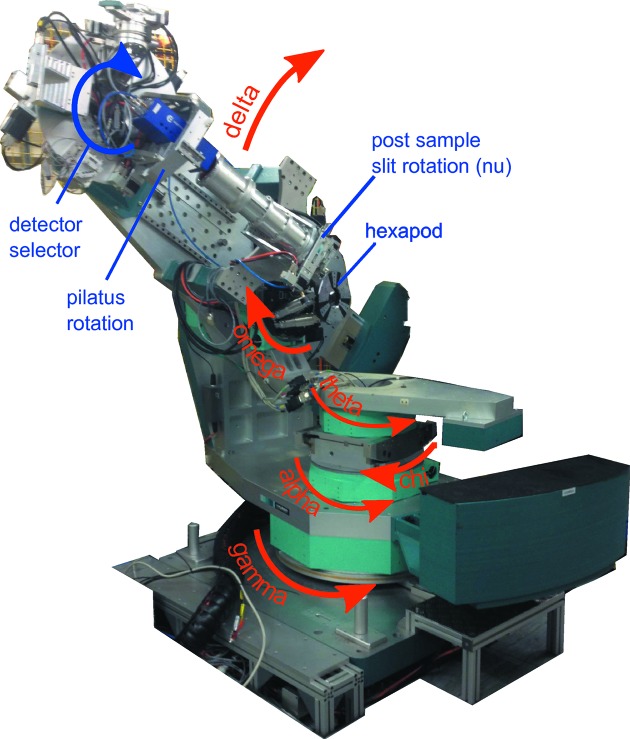
Diffractometer in EH1 showing the unique names of each axis.

EH1 houses a large Huber 2+3 diffractometer (Vlieg, 1998 ▸), shown in Fig. 3 ▸, that can be configured in a horizontal or vertical scattering geometry.

The sample incidence angle and azimuth are given by α and ω for the vertical scattering mode and χ (arc) and θ for the horizontal mode (α table removed). In either scattering geometry a hexapod (Micos) is mounted onto the appropriate azimuthal circle to allow scanning of the sample translations (of the order of ±15 mm) and rotations (of the order of ±10°), for initial alignment. Using a hexapod provides an open arrangement around the sample position enabling quite large and complex experimental chambers to be set up. This has enabled a whole range of user-supplied sample environments to be integrated including an *in situ* spin coater, microwave, high pressures and humidity control. The detectors are mounted on an arm that is decoupled from the sample motions and provides two rotations to enable vertical (δ) and horizontal (γ) motion of the detector while still pointing at the sample. A number of detectors are available (see §2.6), each of which can be moved into position using a rotation stage, that sits behind the diffractometer flight tube. This is a vacuum-pumped pipe with X-ray transparent windows on the front of which are mounted a set of slits to define the line of sight for the scattered X-rays. These slits are mounted on another rotation stage around the sample–detector axis, which acts as the ν rotation for the 2+3 geometry. One of the detector positions, used for a cyberstar scintillator, has another set of slits in front of it also mounted on a rotation axis. These can be software coupled to the post-sample slit rotation to provide a collimated ν rotation that can be used to define the resolution of the surface diffraction measurement, as required for the 2+3 diffractometer implementation. In most experiments, however, an area detector (Pilatus 100K) is used to collect the scattered X-rays. The Pilatus is on an axial rotation stage which is generally used to set the long axis of the detector as either vertical or horizontal depending on the scattering geometry. Measurements are usually made using an open-slit stationary geometry where X-rays form a spot or streak on the detector that can be integrated without the need to perform a rocking curve scan (Schlepütz *et al.*, 2005 ▸).

A large-area detector (Pilatus P2M) is mounted on an independent detector positioning system after the diffractometer. It can be set to give a sample–detector distance of 1.5–3.0 m and vertical and horizontal axes are available to enable the detector to be used for GISAXS measurements with either the straight through beam or with the deflected DCD beam. In addition, a He-filled flight tube with integrated beam stops (for direct beam and reflected beam) is available to reduce the background during GISAXS studies. This flight tube can swing out of the way to enable experiments to use the diffractometer mounted detectors, to cover a wide angular range, whilst the GISAXS is recorded with the large-area detector.

The first hutch is used for a wide variety of other measurements as shown in Table 2 ▸.

#### 2.5.2. Experimental hutch 2   

A vacuum flight tube can be installed in EH1 to produce a beam path to the second hutch. It leads to two sets of monochromatic beam-defining slits, the first set close to the entry point of EH2, while the second set can be positioned as close as possible to the sample. These slits are high precision to accurately define the very small beams that are required to produce a (partially) coherent X-ray beam as required for coherent X-ray diffraction imaging.

**Figure 4:**
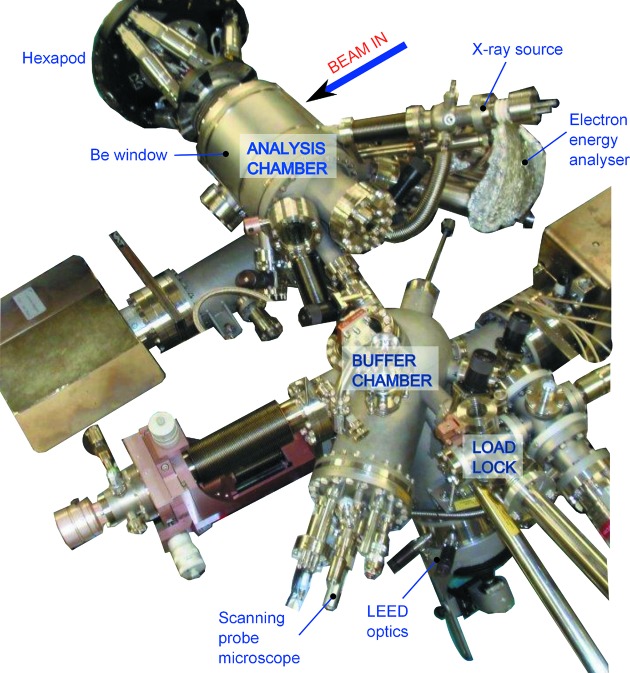
The *in situ* UHV chamber from EH2, with key components labelled.

The second hutch houses a large 2+3 diffractometer oriented to operate with the sample normal horizontal. It is dedicated to the study of *in situ* grown samples in UHV and incorporates a large environmental chamber on the diffractometer (see Figs. 4 ▸ and 5 ▸). It builds on the design of previous chambers that were available on station 9.4 at the Daresbury synchrotron radiation source (*e.g.* Nicklin *et al.*, 1998 ▸). It is a three-vessel system, a turbo-pumped load lock for rapid introduction of samples, a buffer chamber (turbo-pumped and ion-pumped) for sample storage (up to four samples) and preparation and an analysis chamber (turbo-pumped and ion-pumped).

The buffer chamber has facilities for sputtering and annealing, low-energy electron diffraction (LEED) and a scanning probe microscope (Specs, SPM150). The analysis chamber contains a second LEED system and an X-ray source and hemispherical analyser (Specs Phoibos 150) for X-ray photoelectron spectroscopy (XPS). It also has a sputter source for sample cleaning and is able to house up to five evaporation sources for molecular beam epitaxy. It is configured in such a way that the sample always remains in a fixed position at the centre of rotation of the diffractometer. X-rays are directed to the sample through a small Be entrance window (20 mm high by 120 mm long to enable incidence angles of up to 30°). A large Be exit window around the chamber allows the detector to access angles of up to 120° vertically (δ) by 30° horizontally (γ). The chamber mounted Be windows are protected from contamination using internal Be foils that can be removed for cleaning/replacement if required. This configuration allows X-ray reflectivity measurements of up to θ–2θ values of 30° and 60°, respectively. Surface X-ray diffraction measurements tend to use very shallow incidence angles close to the critical angle (typically 0.2° at X-ray energies) and a small horizontal beam size to prevent over-illumination of the sample. Scattering from the entrance and exit Be windows can also lead to significant background, and therefore reducing the entrance slits and exit slits to be as small as possible limits this problem to the lowest angles of δ and γ only. This diffractometer uses a Pilatus 100K area detector for most measurements although it has a similar configuration of detector selection and rotation stages as available in EH1.

### 2.6. Detectors   

The beamline has a number of detectors that are outlined in Table 3 ▸.

## 3. Ancillary facilities   

### 3.1. Preparation laboratories   

The beamline has two laboratories associated with it; the first is set up with a range of chemical preparation and characterization facilities. In addition to standard chemistry apparatus it houses specialist equipment to support or correlate with the beamline measurements including a range of Langmuir troughs (NIMA), a spectroscopic imaging ellipsometer (Nanofilm), a tensiometer (Kruss), a spin coater (Laurell), a small UV ozone cleaning system (Novascan) and differential scanning calorimeter (Perkin-Elmer). A potentiostat that can also be integrated into the beamline control software (Princeton Applied Research) is also available for electrochemistry applications.

The second laboratory houses a UHV chamber with LEED, Auger electron spectroscopy and sample cleaning capability, which is used for sample preparation, evaporator outgassing and source calibration. It also houses a small UHV chamber that can be mounted onto the EH1 diffractometer and a gas panel that allows dosing of well controlled and accurate volumes of gases. A small neighbouring laboratory is fitted out as a clean room and is available for sample mounting or other work requiring a dust-free environment.

### 3.2. Sample environments   

There are several sample environments available at the beamline [including small and large UHV chambers, Langmuir troughs, controlled humidity and temperature (0 to 600°C in controlled gas environment, −100 to 2000°C in vacuum) chambers and solid–liquid cells amongst others] for user experiments, whilst a number of users bring their own cells that either fit into standard goniometers or mount directly onto the hexapod. The geometry of the hexapod means that relatively large sample environments can be mounted; the distance between the hexapod top plate and the sample position is 170 mm and it can support up to 30 kg in any orientation. User cells that have been accommodated include thin film electrochemistry cells, liquid–solid interface cells, vacuum chambers, humidity chambers and hydrostatic pressure cells amongst others. In many cases these have been integrated into the beamline *via* a versatile Eurotherm-based controller for sample temperature control and humidity measurements.

### 3.3. Software   

The beamline is controlled using an architecture based on the Experimental Physics and Industrial Control System (EPICS, http://www.aps.anl.gov/epics/index.php). A graphical synoptic of the whole beamline is produced and windows of specific components can be opened to set and monitor process variables. Users interact with the beamline through *GDA* (*Generic Data Acquisition*, http://www.opengda.org/), a software-based data acquisition system that sits on top of the EPICS layer. The implementation of *GDA* on beamline I07 is driven from the command line, providing a high degree of flexibility in the commands available. Of particular note for I07 is the inclusion of flexible diffractometer calculation software (*Diffcalc*). It enables full reciprocal-space mapping after calculation of an alignment matrix from two or more Bragg peak reflections. The *GDA* software incorporates a scripting interface where commands can be entered together with a fully configured Jython programming environment, enabling very complex scans to be formulated.

## 4. Facility access   

DLS is funded by the UK government *via* the Science and Technology Facilities Council (STFC) and the Wellcome Trust; these funding partners contribute in proportion to their shareholdings which are 86% and 14%, respectively. Non-proprietary beam time, assessed by peer-reviewed application, is free at the point of access for UK academics and funding has previously been allocated under the Framework 7 (EliSA) project for European-led experiments. A modest amount of industrial access has been granted on I07, through a proprietary mechanism and the Diamond industrial liaison group.

## 5. Science highlights   

### 5.1. Surface X-ray diffraction   

SXRD is a powerful technique that allows the arrangement of atoms at the surface of a sample to be determined with very high resolution. SXRD has been used by a number of groups on I07 to study a wide variety of structures including oxide surfaces, thiols on Au(111) and solid–solid buried interfaces (*e.g.* Howes *et al.*, 2013 ▸). The geometry of the scattering and the breaking of the periodicity along the direction of the surface normal leads to streaking in between the Bragg peaks (that originate from the substrate bulk structure). The modulations in the intensity of these features [crystal truncation rods (CTRs)] and the appearance of additional fractional order rods (FORs; that result from the reconstructed surface layers) are due to the surface structure and interference between the bulk and surface scattering. The intensity distribution along these rods can be modelled and is highly sensitive to the ordered surface structure. The use of two-dimensional area detectors has revolutionized the technique, as reflections can now be collected without having to perform a rocking scan, vastly increasing the speed of data collection.

### 5.2. X-ray reflectivity   

The beamline is capable of XRR measurements at low angle (over the critical edge) where details on layer thickness and electron density are key parameters that can be determined and for extended (high angle) X-ray reflectivity where the atomic layer structure can be established (*e.g.* dos Reis *et al.*, 2013 ▸). In XRR, the incident and exit X-ray beams are scanned symmetrically, yielding information purely on the structure along the surface normal. This is a powerful technique even for amorphous films where the detailed shape is related to the film thickness and density as well as interfacial roughness or intermixing.

**Figure 6:**
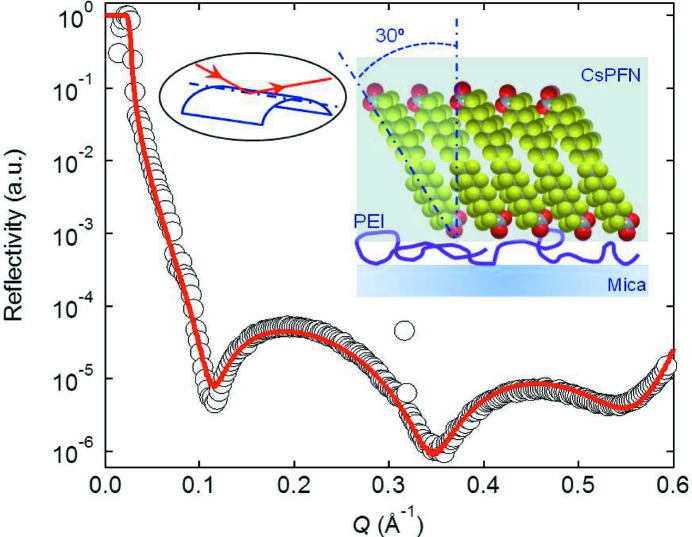
Experimental (open circles) and fitted (solid curve) reflectivity curves for the CsPFN–PEI system at the mica–water interface. The insets show the geometry of the bending mica method on the left, and the possible structure of the polymer–surfactant complex adsorbed on mica on the right.

XRR has been found to be particularly useful for characterization of soft matter structures on mica, as its innate birefringence makes it difficult to apply other commonly used optical techniques (*e.g.* ellipsometry). However, mica has been considered as a non-ideal substrate for XRR measurements, mainly due to the difficulty in achieving flatness over a mesoscopically large area. An experimental setup to overcome this difficulty uses a simple ‘bending mica’ method, where a 30–50 µm-thick mica sheet is slightly bent and clamped over a cylinder of radius 7.5 cm. The enhanced rigidity of the mica sheet along the bending axis ensures a sufficient flatness along the apex of the cylinder (Briscoe *et al.*, 2012 ▸). The bent mica sits inside a liquid cell, allowing information on the interfacial molecular structures of polymers and surfactant layers adsorbed on mica both in air and in aqueous media to be obtained (Speranza *et al.*, 2013 ▸). Fig. 6 ▸ shows an example result for a complex of a fluorinated surfactant, CsPFN, and a positively charged polymer, PEI.

### 5.3. Grazing-incidence small-angle X-ray scattering   

GISAXS is becoming increasingly useful to yield information about the shape, size distribution and interparticle correlations of nanostructures on surfaces or in a thin film. The analysis of GISAXS data is not yet as mature as for conventional SAXS data, but it can still reveal a lot of information about the structures. The position and size of the features are related to the size and characteristic separation of the nanostructures, while features such as flares in specific directions can reveal faceting of the nanoparticles. GISAXS at I07 has been applied to several systems and has proven especially useful for looking at soft-condensed matter polymer structures or molecular crystals (*e.g.* Prehm *et al.*, 2011 ▸).

### 5.4. Grazing-incidence wide-angle X-ray scattering   

**Figure 7:**
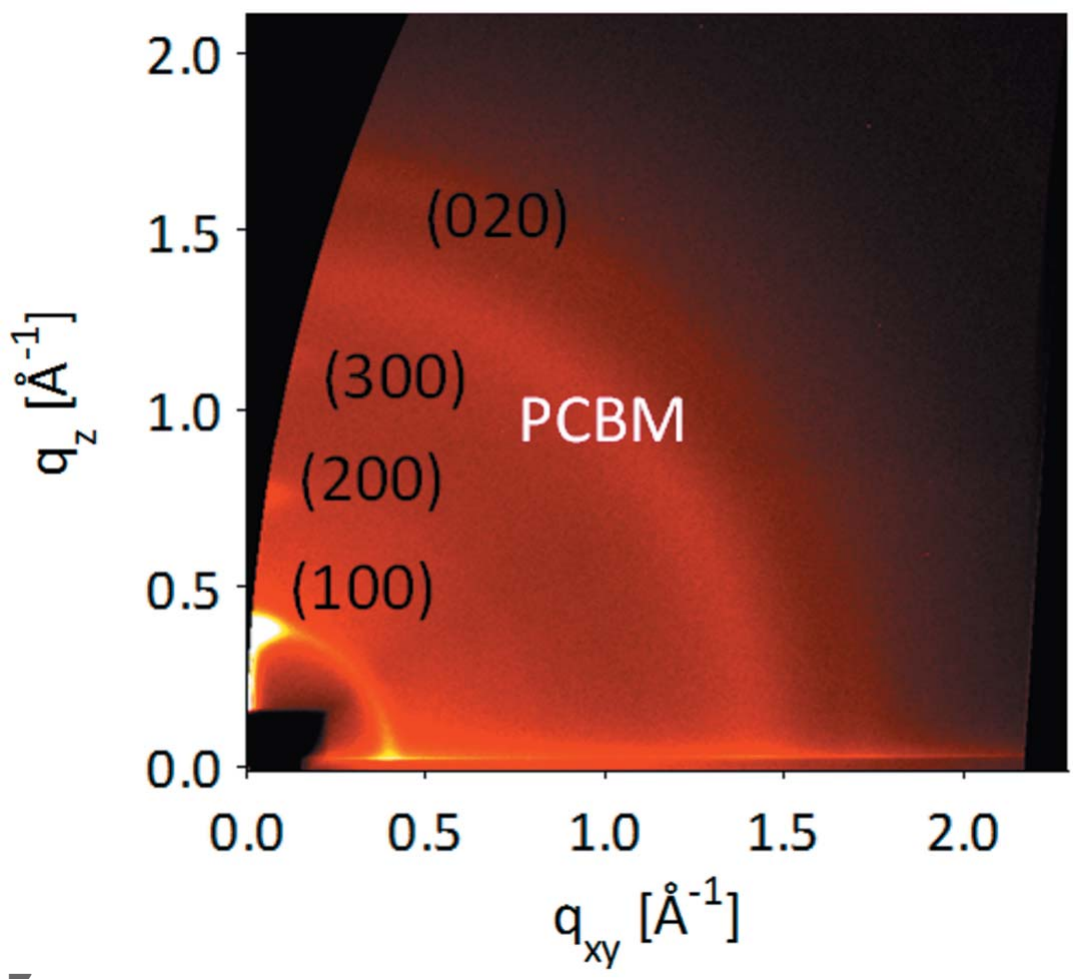
GIWAXS image of a P3HT/PCBM polymer blend, corrected for geometrical effects of the flat detector.

In grazing-incidence wide-angle X-ray scattering (GIWAXS) mode, the large-area detector can record the scattering over a wide angular range (∼25° vertically and horizontally). Good statistics can be collected in exposures of typically 5 s whilst most features are visible in ∼0.5 s exposures. Therefore, this mode of operation is particularly suited to dynamic measurements where structural changes such as developing strain or changing crystallinity can be followed during sample processing (*e.g.* thermal treatment). It has proven to be particularly popular at I07 in the study of organic photovoltaic structures (*e.g.* Agostinelli *et al.*, 2011 ▸). Fig. 7 ▸ shows a single image recorded for a blend of poly(3-hexyl)thiophene (P3HT) and [6,6]-phenyl C61 butyric acid methyl ester (PCBM), where the crystalline P3HT features appear as short streaks while the amorphous PCBM results in a broad diffuse ring of scattering. The development of strain and average domain size was determined during annealing and related to an optimum annealing strategy to form good quality heterojunction devices.

### 5.5. Liquid surface X-ray scattering   

Beamline I07 uses a DCD system (Arnold *et al.*, 2012 ▸) to deflect the X-ray beam through Bragg scattering by dissimilar crystals. A recent upgrade now makes use of InSb(111) and InSb(220) to produce a net deflection of the beam, that is directed to the centre of the diffractometer. Rotating the whole crystal deflection assembly around the incoming beam results in a changing incidence angle on a sample that remains horizontal. The scattered X-rays can be detected by setting the diffractometer detector rotations (δ and γ) appropriately to enable either the X-ray reflectivity or the grazing-incidence diffraction (using a pinhole geometry) to be recorded. It has been used extensively for looking at thin lipid films and how molecules interact with the self organized films (*e.g.* Watkins *et al.*, 2014 ▸; Clifton *et al.*, 2012 ▸; Hemming *et al.*, 2015 ▸). The implementation at I07 allows the energy to be tuned up to 30 keV and it has therefore also been used to study liquid–liquid scattering.

### 5.6. Coherent X-ray diffraction (CXD)   

The Diamond synchrotron source produces X-rays with a very high coherence length. Although not fully optimized for coherent X-ray diffraction, the I07 beamline can be used to study the CXD from particles of approximately 300 nm in diameter. I07 has been used in this mode by several groups and one highly desirable feature has been the ability to combine the CXD with the *in situ* UHV capabilities offered by the chamber in EH2. The coherent X-rays are produced by closing the slits down to ∼10–20 µm (order of the coherence length) and the scattered X-rays are detected on the Pilatus 100K detector that is placed typically at a distance of 1.5 m from the sample. This particle size and detector distance with the 172 µm pixels gives the correct amount of oversampling to enable reconstruction of the particle shape and strain which are related to the amplitude and phase of the recovered signal, respectively. *In situ* alloying has been studied by Xiong *et al.* (2014 ▸) who were able to observe how depositing copper on top of gold nanoparticles, together with subsequent annealing, could lead to details of the dynamics of the process with copper entering through channels in the nanoparticles. In particular, phase loops were observed indicating the high degree of strain that occurred at the corners of the faceted gold particles.

### 5.7. Time-resolved studies   

Many studies on beamline I07 have made use of the high flux of X-rays and the fast speed at which the detectors can run to monitor dynamic processes. These generally occur on sub-second time scales, and have included phase changes during annealing or during charge/discharge cycles in rechargeable battery materials (Roberts *et al.*, 2014 ▸). In this case a cell was designed to enable depth profiling through changing the incidence angle and fast acquisition with the small Pilatus 100K, that was set at a specific angle to observe peaks from the two phases present in the charging system. In this mode the point at which the phase switched and how it was distributed throughout the material could be tracked. The reversibility of the process and the effect of charging rate were reported.

### 5.8. Multi-technique UHV studies   

**Figure 8:**
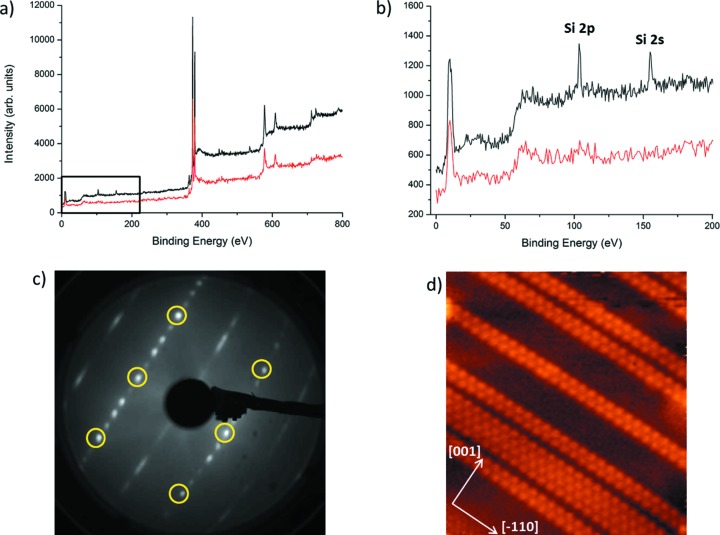
Different techniques measured *in situ* in the same chamber for studying a sample of silicon nanoribbons grown on an Ag(110) substrate. (*a*) XPS spectra showing signals collected for clean Ag(110) (red line) and after <1 monolayer Si deposition. (*b*) XPS close-up view labelling the additional peaks due to Si. (*c*) Low-energy electron diffraction pattern showing a (5 × 2) superstructure. The pattern was collected at 70 eV and the integer order spots are highlighted with yellow circles. (*d*) STM image showing the characteristic nanoribbon structures [200 nm × 200 nm, *V*(*t*) = 1 V, *I*(*t*) = 50 pA].

Several experiments have made use of the multiple techniques available on the same chamber in EH2 to fully understand the sample. The chemical nature can be established using XPS, whilst the physical structure can be investigated by LEED, scanning tunnelling microscopy (STM) and SXRD. Here we show how these techniques have all been used to study an ordered surface reconstruction when silicon is deposited on Ag(110) at elevated temperature (280°C) and with a coverage of less than one atomic monolayer. Fig. 8 ▸ shows the (5 × 2) reconstruction (easily observed in the LEED pattern) that is formed by nanoribbon features on the surface (as seen in the STM image). The XPS reveals that there is no contamination and only silicon and silver are present on the sample.

**Figure 9:**
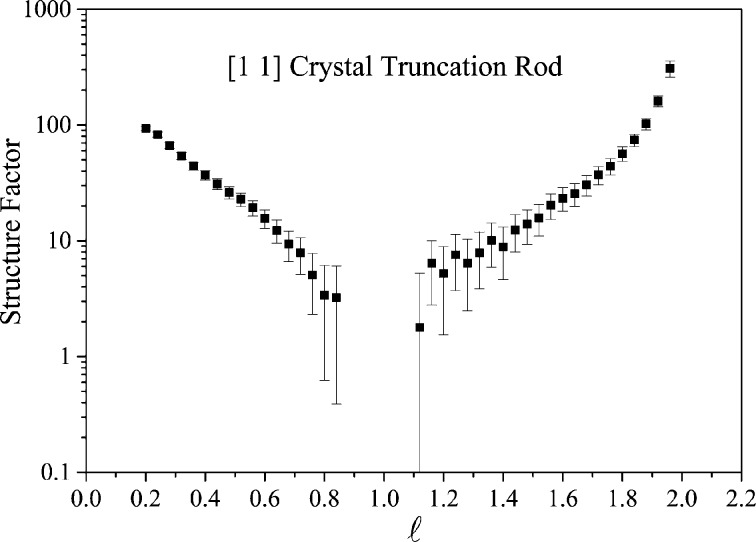
CTR measured from a sample grown *in situ* in the EH2 UHV chamber.

The sample was then aligned for SXRD measurements and a large number of CTRs and FORs were recorded; a typical CTR is shown in Fig. 9 ▸. This reconstruction is complex, requiring as much information as possible to try to fully understand the restructuring of the interface. The correlation of the techniques in the same chamber is invaluable in cases such as these.

## 6. Summary   

Beamline I07 at Diamond Light Source is a versatile high-resolution diffraction facility, with diffractometers and endstations configured to be particularly useful for surface and interface studies. It can provide information on the atomic structure of surfaces and interfaces as well as about the shape and size of nanostructures at an interface. State-of-the-art detectors and the high X-ray flux enable low-noise and dynamic studies to be carried out, making the measurements available to an ever increasing variety of complex samples.

## Figures and Tables

**Figure 5:**
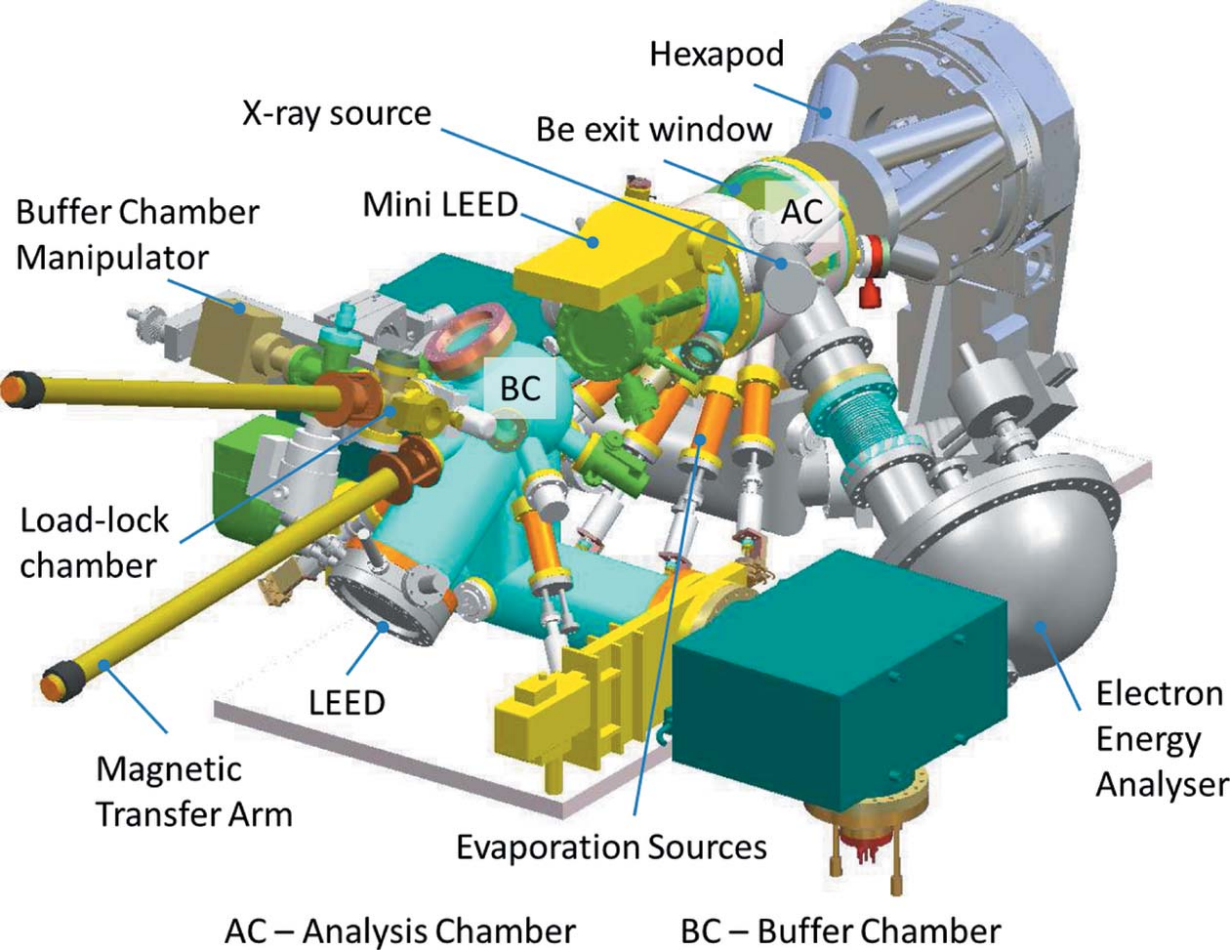
Schematic outline of the UHV chamber from EH2.

**Table 1:** Positions of additional beamline components in the optics hutch

Component	Distance from source (m)	Use	Notes
Collimators	22.0, 24.4, 28.7	Blocks Brehmstrahlung	Three collimators to allow only synchrotron beam to sample
Beam shape and intensity monitors	23.6, 33.1	Image beam and measure intensity	Camera images beam on screen, photodiode detects direct or scattered X-rays
Quadrant beam position monitors	28.0, 32.6	Position and intensity of beam	After monochromator and mirrors
Calibration foils	32.9	Energy calibration	Different foil materials to calibrate monochromator
Shutter	33.5	Final shutter	Essential part of personnel safety system

**Table 2:** Additional techniques available in EH1 of beamline I07

Experiment	Details	Sample–detector distance	Comments
GISAXS	P2M mounted on *XYZ* table	1.5–3.0 m	Flight tube with integrated direct beam and reflected beam stop
GIWAXS	P2M mounted on diffractometer	30–50 cm	Large area detected in single snapshot
XRR	θ–2θ scans using pilatus 100K	900 mm	Multiple regions of interest to measure signal and background simultaneously
Liquid XRR	Use DCD and track reflected beam with diffractometer	900 mm	Deflects X-rays; no movement of the sample

**Table 3:** Detectors available on I07 with their main uses outlined

Detector	Main use
Cyberstar scintillator	X-ray reflectivity, surface X-ray diffraction
Avalanche photodiode	Photodiode with high dynamic range
Pilatus 100K	Surface X-ray diffraction, X-ray reflectivity and grazing-incidence diffraction (172 µm pixel size)
Merlin Quad (2 × 2)	Small pixels (50 µm) area detector; total active area 28 mm × 28 mm
Pilatus 2M	GISAXS and GIWAXS. 172 µm pixel size; total active area 254 mm × 289 mm
